# The Improvement Path for Regionally Coordinated Green Development: Evidence from Social Network Analysis

**DOI:** 10.3390/ijerph191811703

**Published:** 2022-09-16

**Authors:** Yang Zhou, Hankun Wang, Zuqiang Wang, Xiang Dai

**Affiliations:** 1 The Party School of Zhejiang Provincial Committee of the Communist Party of China, Hangzhou 311121, China; 2 Zhejiang Research Center of Xi Jinping Thought on Socialism with Chinese Characteristics for a New Era, Hangzhou 311121, China; 3 Zhejiang Provincial Party School Research Center for Comprehensively Strictly Governing the Party, Hangzhou 311121, China; 4 Department of Public & International Affairs, City University of Hong Kong, Hong Kong 999077, China; 5 College of Business and Economics, Australian National University, Canberra, ACT 2600, Australia

**Keywords:** environment regulation, social network analysis, spatial nonlinearity effects, regionally coordinated green development

## Abstract

Regionally coordinated green development has been widely documented in China. However, most previous studies have investigated it from the perspective of linearity, while the spatial correlation of green development is nonlinear. Based on 48 cities in Bohai Rim, this study used a social network analysis to measure the spatial network, with an emphasis on the internal structure of regional green development, and analyzed the driving factors of regionally coordinated green development from the perspective of nonlinearity. We found that large cities have formed a “siphon effect” and that the polarization of eco-efficiency has become increasingly serious. There are limited connections, some of which are redundant, in the spatial network of green development, while the stability of the network is strong. Additionally, reducing the differences in environmental regulation approaches among cities can have a positive impact on the spatial correlation and spillover effect of green development, thereby promoting regionally coordinated green development among cities in the Bohai Rim.

## 1. Introduction

Since the 18th National Congress of the Communist Party of China, the characterization of the development of the Chinese economy has changed from rough development to sustainable development. However, increasing environmental pollution and extensive resource utilization have severely restricted the overall modernization process. The 5th Plenary Session of 19th Central Committee of the Communist Party of China further emphasized that China must adhere to green development goals and create a harmonious, coexisting relationship between humans and environment. The report of the 19th National Congress of the Communist Party of China proposed the regionally coordinated development strategy. Therefore, the rational layout of green industries, the coordination of the green development of various regions, and the improvement in the efficiency of resource utilization have great significance in the high-quality development of a green economy.

Due to differences in resource endowments, geographical locations, economic foundations, and development strategies and guidelines, Chinese green development processes show large regional differences and serious polarization. Guided by the goal of ecological civilization construction, all regions are continuing to explore new green development paths. Although regional green development with a focus on urban agglomerations has achieved certain results, the problems posed by regional imbalances in green development remain prominent, and the situation is troubling. For this reason, the “Overall Plan for Ecological Civilization System Reform” issued by Chinese government proposes an enhancement of the integrity and coordination of the reform of the ecological civilization system. Regionally coordinated green development advocates for industrial-level green development, with endowments to various regions, and promotes a new, integrated green development pattern; this builds an efficient, comprehensive, open, and shared green development system by integrating the resources of various cities in the region. It is conducive to the overall improvement of green development.

The results of the current research, which surround the theoretical connotations, measurement methods, and implementation paths for green development, are very fruitful; however, room for improvement remains in regionally coordinated green development. Based on the inherent requirements of China’s real-world development, this research focuses on regionally coordinated green development. This analysis is achieved through empirical measurements of China’s urban-level, coordinated green development capabilities and systematic analyses of the internal evolution mechanism and external driving mechanism of coordinated green development. This study provides a useful, decision-making reference point for the realization of the long-term goal of a “Beautiful China”.

## 2. Literature Review

Eco-efficiency measurement methods assess productivity under environmental restraints and have been widely used to evaluate the performance of green development approaches. Eco-efficiency measurement methods were first introduced by Schaltegger and Sturm [1] and have become one of main topics in research of green development approaches in recent years. The ratio approach [2,3], the material flow analysis (MFA) approach [4,5], and the data envelopment analysis (DEA) approach [6,7] are often applied for evaluating eco-efficiency. In the literature, studies on eco-efficiency can be divided into two main aspects: factors influencing eco-efficiency and spatial correlations of eco-efficiency. In terms of influencing factors, high GDP per capita, well-organized industrial structures, effective innovation abilities, high population densities, and developed environmental awareness of a given population have been documented to have a positive effect on eco-efficiency. In addition, Liang and Yang [8] found that urbanization has a significant effect on the eco-efficiency of a given area; the relation between urbanization and eco-efficiency is an inverted U curve. Zhu et al. [9] showed that eco-efficiency is positively affected by green technology, while innovation costs can be a burden and can reduce eco-efficiency. Dasgupta et al. [10] suggested that environmental policy, monitoring, and enforcement activities can have a positive influence on the environmental performance of polluters.

Spatial correlation, in relation to eco-efficiency, has also been widely investigated by scholars recently. Using Markov chain, Pan et al. [11] studied the club convergence of eco-efficiency in China, and they believe that the spatial connections among regions may come from the spatial connections between regional characters. Li et al. [12] applied a spatial econometric model to find the spatial connections influencing eco-efficiency in China. Li et al. [13] concluded that there is a negative relation between urbanization and energy efficiency; they found that this negative relation is directly influenced by the negative effects of key provinces and is indirectly influenced by the positive spillover effect of neighboring regions. The spatial connections influencing eco-efficiency have been discussed extensively in the literature. However, the majority of these studies discussed the spatial connections of eco-efficiency in a linear way [14,15]. In contrast, the spatial connections influencing eco-efficiency are likely to be nonlinear and modelling them in a linear way may cause biased policy-making decisions. Therefore, this paper fills this gap, as we used a social network analysis to graph the nonlinear spatial connection of green development approaches.

Some scholars investigated the integrated green development in China and most of them supported the idea that integrated development would enhance green productivity efficiency. Yang et al. [16] suggested that cooperation between developed and undeveloped regions in the development of technology would lead to a reduction in ineffective capital investment and would improve economic output efficiency. Cui and Li [17] studied the efficiency of the airline industry and posited that cooperation between airline firms would enhance their efficiency. If all airline companies were in a cooperative relationship, then their efficiency would be improved in comparison with a scenario where all airline companies are in competitive relationship. In addition, Luo and Shen [18] indicated that the efficiency of cooperation between cities depends on the mechanisms, nature, and scope of their cooperation, the selected partners, and the roles of the actors in the partnership. There are also some scholars who have used the coupling coordination degree approach to study the degree of coupling coordination between the subsystems of economy, ecological environment, urbanization, and food [19,20,21,22]. However, the potential factors with impacts on regionally coordinated green development have not yet been discussed. Therefore, this paper will use a quadratic assignment procedure (QAP) to explore the driving factors of regionally coordinated green development approaches among cities in the Bohai Rim.

In the literature, most studies have approached the spatial correlation of green development in a linear way. However, considering that connections between regions are complicated, the spatial connections of green development approaches should be approached in a nonlinear way. Moreover, in the past, the central government of China has assigned great importance to the integrated development of the Bohai Rim area and believe it will reshape the industry distribution and alleviate the environmental problem in the Bohai Rim area. However, there has been little research about whether this is applicable. In addition, as the government is increasingly interested in regionally coordinated green development, there is some evidence that green development remains uneven across China’s regions; however, little is known about the “how, when and why” of the occurrence of such progress. To fill the above research gaps, this paper makes the following contributions.

Firstly, this paper uses a window–super-slack-based measure to calculate eco-efficiency, indicating the level of green development in each city in the Bohai Rim area. Additionally, we measured the degree of regionally coordinated green development using a gravity model. The strength of the spatial correlations can quantify the spatial correlation of green development among cities in the Bohai Rim area, which can reflect the ability of cities to have and receive influence surrounding green development from other cities. Secondly, this paper focuses on a spatial network analysis of green development, which shows the internal characteristics and mechanisms of the green-development-related spatial correlation and spillover effect. Thirdly, this paper provides support for the regionally coordinated green development of the Bohai Rim area and discusses the applicability of it through a QAP, which was used to explore the driving factors of regionally coordinated green development, providing empirical support for the integrated development policy. This study has practical significance for policymakers in formulating policies scientifically and can improve the effectiveness of policy implementation in regionally coordinated green development.

This paper is organized as follows: Section 3 presents the data and gives a brief description of the methods used in this paper. Section 4 addresses the spatial network of regional green development and illustrates the factors that potentially affect regionally coordinated green development. Section 5 provides our conclusions and policy suggestions.

## 3. Methods and Data

### 3.1. Measuring the Spatial Correlation of Eco-Efficiency

A DEA posits no requirement for the shape of the production function for inputs and outputs and is able to evaluate complex production relations. If there are multiple decision-making units (DMUs) in the frontier, then the traditional slack-based measure (SBM) fails to determine the differences among the DMUs. Aiming to solve this problem, this paper used an adjusted SBM [23] and brought the undesirable outputs into consideration. In addition, to facilitate an analysis of the dynamic spatio-temporal evolution of the measured eco-efficiency, this study combined a window analysis with a super undesired SBM model. Based on the length of the research in this paper, the number of windows was determined to be 3, and each window was calculated to be 3 n. The efficiency value of each DMU and the average value of each DMU at each time point was calculated as the eco-efficiency value of the DMU.

For efficient DMU_*k*(*x_k_*, *y_k_^d^*, *y_k_^ud^*), with the possible production set of {(*x*, *y*), *x* ≥ ∑_(*j*=1,*j*≠*k*)_*^n^*, *x_ij_* *λ_j_*, *y* ≤ ∑__(*j*=1,*j*≠*k*)_*^n^*, *y_rj_ λ_j_*}, $\left(\bar{x},\bar{y}\right)$ is the projection of the evaluated DMU*_k_* in the model.

(1) $$minEE=\frac{\frac{1}{m}\sum_{i=1}^{m}\frac{{\bar{x}}_{i}}{x_{ik}}}{\frac{1}{\left(q_{1}+q_{2}\right)}\left(\sum_{r=1}^{q_{1}}\frac{{\bar{y}}_{r}^{d}}{y_{rk}^{d}}+\sum_{t=1}^{q_{2}}\frac{{\bar{y}}_{t}^{ud}}{y_{tk}^{ud}}\right)}$$

Which is subject to:

$$\sum_{j=1,j\neq k}^{n}x_{ij}\lambda_{j}\leq {\bar{x}}_{i}$$

$$\sum_{j=1,j\neq k}^{n}y_{rj}^{d}\lambda_{j}\geq {\bar{y}}_{r}^{d}$$

$$\sum_{j=1,j\neq k}^{n}y_{tj}\lambda_{j}\leq {\bar{y}}_{t}^{ud}$$

$$\bar{x}\geq x_{k},\;{\bar{y}}^{d}\leq y_{k}^{d},\;{\bar{y}}^{ud}\geq y_{k}^{ud}$$

$$\lambda ,s^{-},y_{r}^{d},y_{r}^{ud}\geq 0$$

where eco-efficiency (*EE*) is the measured super efficiency and can be larger than 1.

After the measurement of eco-efficiency, we constructed the green development network for the Bohai Rim area. Each city in the Bohai Rim area was regarded as a “point” in the network, and the spatial connections of eco-efficiencies formed the “line” between each point. In the literature, the correlation for the spatial perspective of eco-efficiencies between two regions has been measured by the VAR model. However, the VAR model fails to reflect the dynamic change in the network and is very sensitive to the lag effect of the variables. Therefore, this study used a modified gravity model, as follows:

(2) $$R_{m,n}=\frac{K_{m,n}\sqrt[{3}]{P_{m}G_{m}EE_{m}}\sqrt[{3}]{P_{n}G_{n}EE_{n}}}{D_{m,n}^{2}},K_{m,n}=\frac{EE_{m}}{EE_{m}+EE_{n}},D_{m,n}=\frac{d_{m,n}}{g_{m}-g_{n}}$$

where *R_m,n_* represents the spatial correlation between city *m* and city *n*; *P_m_* and *P_n_* are the populations of city *m* and city *n*, respectively; *G_m_* and *G_n_* are the GDPs for city *m* and city *n*, respectively; *EE_m_* and *EE_n_* are the eco-efficiencies of city *m* and city *n*, respectively; *K_m,n_* shows the ratio of eco-efficiency of city *m* to the sum of eco-efficiency of city *m* and city *n*; *D_m,n_* is the economic distance between city *m* and city *n*; *d_m,n_* is the geographic distance between city *m* and city *n*. *g_m_* and *g_n_* are the GDP per capita for city *m* and city *n*, respectively. By the calculation, the correlation between the eco-efficiencies of cities in the Bohai Rim area form the following matrix: [*R_m,n_*]^48×48^.

### 3.2. Social Network Analysis

To measure the inside structure of the network in our study, we used a social network analysis. The social network analysis consisted of two major parts: a centrality analysis and a block model analysis. For the centrality analysis, we used a degree centrality measurement, a closeness centrality measurement, and a betweenness centrality measurement to measure the roles and effects of the various regions in the eco-efficiency network. The degree centrality measurement was used to represent the beneficial between-region correlations inside the eco-efficiency network. The degree centrality measurement was divided into an in-degree centrality measurement and an out-degree centrality measurement. In terms of the in-degree centrality measurement, a larger number indicates that the region in question has a greater correlation with other regions in the eco-efficiency network. As for the out-degree centrality measurement, a larger number indicates that the region in question poses a greater spillover effect on other regions in the eco-efficiency network. The closeness centrality measurement determines how simple it would be for a region to develop an internal connection with other regions in the eco-efficiency network, with each region playing a central actor role. The betweenness centrality measurement is used to determine which region will play the intermediary role (or the “Bridge” role) in the eco-efficiency network.

The block model analysis involves conducting a spatial cluster analysis for the whole network; this analyzes the connections between each group and provides more intuitive insight into how each region is connected in the whole network. This method first divides regions into different plates and then studies the spillover effect between each plate. Using block model analysis, the internal structural status of the network can be revealed. This analysis is especially useful when there are potential clusters in the network.

### 3.3. Quadratic Assignment Procedure (QAP)

Due to contradictory assumptions surrounding independent variables, traditional studies using econometric methods to analyze the spatial spillover effects of eco-efficiency often show errors, such as biased estimation results; meanwhile, the QAP method can overcome these potential biases. Since the QAP method is based on the permutation of the matrix, the correlation coefficient of the two matrices is given by comparing the similarity in the lattice values of the two square matrices, and the nonparametric test is carried out using the given coefficients. The QAP method does not require an assumption that the independent variables are opposed to each other, and the test results are more robust. Therefore, this study adopts the QAP method to analyze the spillover effects and potential spatial drivers of eco-efficiency. The specific model is as follows:

ER = F (GD, ED, TD, UD, ID, EPD)

where ER represents the two-value network matrix of the correlation relation of regional eco-efficiency. More details on variable descriptions are in Table 1.

### 3.4. Data and Sources

Considering the accessibility and availability of data, this study exploited the annual data of 48 cities in Bohai Rim from 2005 to 2017. Most data came from the China Urban Statistical Yearbook, China Environmental Statistical Yearbook. Table 2 shows the indicator system used in super SBM models for measuring eco-efficiency. Labor, resources, and capital investment were selected as inputs and energy uses in industrial built-up areas—i.e., electricity, liquefied petroleum gas, and water consumption—were considered as resource input indicators. The formula used was Kt = Kt − 1 (1 − δ) + It, where K indicates the capital stock, ang I indicates the investment in the current year, δ_i_ indicates the depreciation rate, and i and t indicate the cities and years, respectively. As in [24], 2005 was selected as the starting value and 9.6% was the annual depreciation rate. In terms of outputs, the constant GDP of 2005 was used as the expected output and three industrial wastes were used as the undesired output. Table 2 shows the explanation and measurement for the variables used in the spatial correlation analysis.

## 4. Results and Discussions

**Figure 1:**
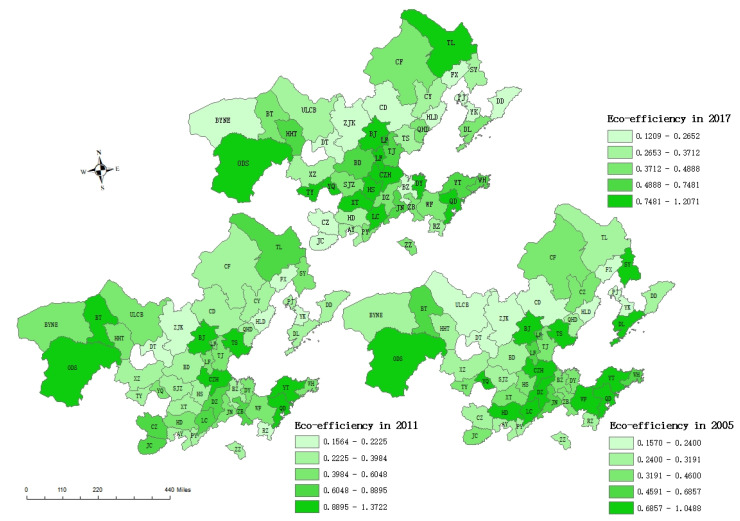
Distribution of eco-efficiency inside Bohai Rim in 2005, 2011, and 2017.

The eco-efficiencies of 48 cities were calculated using the adjusted SBM model. We found that eco-efficiency increased over time, and it coincided with the raising environmental consciousness in China. Additionally, Beijing, Cangzhou, Ordos, Qingdao, and Yantai all showed high eco-efficiency from 2005 to 2017; meanwhile, Rizhao, Zaozhuang, Anyang, Puyang, Huludao, Chaoyang, and Fuxin showed consistently low eco-efficiency. Additionally, Weihai, Yangquan, Handan, Xinzhou, and Tangshan showed fluctuating eco-efficiency performance. In addition, the eco-efficiency in the Bohai Rim area was found to be unstable over time. Among the 48 cities in the Bohai Rim area, there we found that less than one quarter of cities had good eco-efficiency performance. The difference in eco-efficiency levels among cities was found to be significant. We found that cities showing high eco-efficiency scores usually had good transport systems and strong economic development, such as provincial capitals or coastal cities. Additionally, the cities that had low eco-efficiency scores were usually the cities with exhausted nature resources. We found a cluster phenomenon among the geographical locations; this adjusted with the passage of time, which is consistent with previous papers [25,26,27]. Furthermore, areas with high eco-efficiency were found to be consistently concentrated in the eastern coastal areas. We found an expansion trend during the period of 2005–2017, which formed a contiguous belt from east to west. Overall, a significant spatial difference was found in the eco-efficiency of cities in the Bohai Rim area, with a distribution pattern of “strong in the east and weak in the west” (Figure 1). Although the eco-efficiency of inland cities has showed a gradual improvement over time, a problem of uncoordinated green development in the Bohai Rim area remains.

### 4.1. Social Network Analysis of Eco-Efficiency

Through a social network analysis, we found that the actual eco-efficiency correlation among cities in the Bohai Rim area was 527 in 2017, while the maximum correlation among all 48 cities in the Bohai Rim area was 2256. The network density was 0.234, meaning that only 23.36% of eco-efficiency associations can be observed. This indicates that the eco-efficiency associations among cities in the Bohai Rim region are low; thus, the capacity for regionally coordinated eco-efficiency development still has potential for improvement. From Table 3, we observe that the network correlation is always 1, indicating that there is no isolated city in the eco-efficiency network; each city in the Bohai Rim area has a spillover effect on other cities and will be influenced by other cities. Additionally, from Table 3, we observe that the network hierarchy is almost always 0.04. This shows that all cities were ranked at the same level and no city holds a superior position. Cities with different ecological efficiencies can influence each other, and the stability of the eco-efficiency network is relatively high. Additionally, the network efficiency was 0.71 in 2017. This indicates that the influence of cities on their neighbors—the overflow of eco-efficiency from spatial perspective—has an important multiple-superposition phenomenon. The associated eco-efficiency network is stable, but it shows many redundant connections. In summary, while the stability of the eco-efficiency network is not weak, the connections among cities in the Bohai Rim area are limited, with some redundant connections. The regional coordination of green development remains a primary task for the Chinese government.

#### Centrality Analysis

Table 4 shows the degree centrality, closeness centrality, and betweenness centrality measurements of the eco-efficiency network in the Bohai Rim area. As we can see from Table 4, the mean of the out- and in-degree centralities for every city in the Bohai Rim area is 10.98, and there are 14 cities with higher-than-average out- and in-degree centralities. The out- and in-degree centralities of Dongying, Ordos, Qingdao, Yantai, Weihai, and Zibo are relatively high. These cities are economically developed or eastern coastal cities with convenient transportation; additionally, they possess a good ability to both influence and receive influence from other cities in the eco-efficiency network. Hence, Dongying, Ordos, Qingdao, Yantai, and Zibo were found to be in the center of the spatial eco-efficiency network in the Bohai Rim area, and each showed a close connection with other cities. However, Ulanqab and Bayannaoer had relatively low out- and in-degree centrality and were in a marginal position of the eco-efficiency network.

The mean of the closeness centrality measure was 80.5, with a maximum of 106 and a minimum of 54, indicating an overall balance. Additionally, we found that the closeness centrality measure was higher than 70 for the majority of cities, except for Dongying, Ordos, Qingdao, Yantai, Weihai, Jinan, and Zibo. Considering that the closeness centrality measure represents the ease of transport to other cities in the network, the whole eco-efficiency network is centered in those cities. Roughly speaking, due to great economic development, widely connected transportation, and advanced technology, the eastern coastal cities showed a strong connection with other cities in the eco-efficiency network. However, inland cities were found to have weaker and fewer connections with other cities because of the relatively minimal economic development and inconvenient transportation connections.

The mean of the betweenness centrality measure was found to be 16.75, with a maximum value of 146.17 and a minimum value of 0. From Table 4, the betweenness centrality measurements of Dongying and Ordos were 146.17 and 108.53, respectively, which were much higher than those for the other cities in the Bohai Rim area. These results indicated that these two cities are the main intermediary or “bridge” cities in the spatial eco-efficiency network. In contrast, the betweenness centrality measure of Bayannur City was zero, which shows that it has no control over the eco-efficiency linkages of other cities.

This may be because Bayannur City’s own eco-efficiency is low, its economic level is underdeveloped, and it is unable to form a correlation with other cities in terms of green technology development and environmental regulation, etc.; additionally, its ability to influence and be influenced by other cities is poor. Overall, it can be seen that, within the Bohai Rim region, Dongying and Ordos are the center points of eco-efficiency development; additionally, the spatial linkage and spillover effects of eco-efficiency growth have been formed especially in inland areas and eastern coastal areas.

### 4.2. Block Model Analysis

In the previous section, we demonstrated that different cities have different performances in the centrality analysis. Specifically, we found that eastern coastal cities and cities with high GDP play an important role and may work as the center or “bridge” in the network structure of eco-efficiency. To further analyze the internal characteristics of the spatial network of eco-efficiency across cities, the present section adopts a block model analysis. According to the literature, where the maximum segmentation depth is limited to 2 and the maximum concentration is limited to 0.2, the 48 cities in the Bohai Rim area were separated into 4 blocks, as shown in Table 5. From the map, there is no obvious geographical division between the plates. Additionally, the cities in each plate cannot be simply divided into the southern, central, and northern Bohai Rim, which supports the practicability of the social network analysis of the eco-efficiency network in the Bohai Rim area.

The eco-efficiency correlations among the plates in the Bohai Rim area are displayed in Table 6. In the spatial eco-efficiency network in the Bohai Rim area, there are 88 (35 + 30 + 8 + 15) connections within each plate, which account for 16.69% of all connections in the eco-efficiency network. Additionally, there are 439 (123 + 33 + 84 + 199) connections between the plates, which account for 83.31% of all connections in the eco-efficiency network. By comparing the connections within and between each plate, spatial correlations and spatial spillover effects can be found among the eco-efficiencies of the cities, and the spillover effects among the plates hold more significance.

For the first plate, there are 35 internal relationships, 123 overflow relationships that receive influence from other plates, and 182 overflow relationships that influence other plates. There are more overflow relationships which influence other plates than those that receive influence. Additionally, the expected internal relationship ratio (42.55%) is higher than the actual internal relationship ratio (16.13%). Therefore, the first plate is regarded as the “Net overflow plate”, which includes 21 cities, such as Anyang, Bayannaoer, and Baoding. The second plate is the “Agent plate”, including 13 cities, such as Chaoyang, Chifeng, and Dandong. In this plate, the total number of internal relationships is 30, and the overflow relationships (33) of influence received from other plates are fewer than the total spillover relationships influencing other plates (97). Additionally, the actual internal relationship ratio (23.62%) is close to the expected internal relationship ratio (25.53%). The third plate is in the “Two-way overflow plate” class, and includes Tianjin, Taiyuan, and Hohhot, etc., and this plate receives influence from other plates and poses influence on other plates in equal measure. Most cities in this plate are provincial capital cities and normally have high economic development, advanced green technology, and convenient traffic connections; therefore, these cities are more likely to have a significant impact on the cities of other plates. For the fourth plate, there are more overflow relationships of received influence (199) than there are spillover relationships of influence on other plates (93). The actual internal relationship ratio is 16.96%, which is higher than the expected internal relationship ratio (14.90%); therefore, the fourth plate is considered to be the “Main benefit plate”.

In order to further analyze the connections inside the eco-efficiency network, we used Ucinet to generate density matrices between each plate. The density of the whole eco-efficiency network was found to be 0.23. Therefore, when the value of a given plate’s density is bigger than 0.23, the value should be 1 or 0. Using this method, we were able to obtain an image matrix for every plate. From Table 7, the first plate plays the role of an “engine” in the spatial eco-efficiency network, passing the kinetic energy of eco-efficiency growth to the “Two-way overflow plate” and the “Main benefit plate”. Most cities in the first plate are concentrated in the central region. Holding an “intermediary” and “bridging” role, the second plate can pass the kinetic energy of eco-efficiency growth to the “Main benefit plate”. Most cities in the second plate are in the northeastern region. The cities in the two-way spillover plate are mostly provincial capital cities, which usually have better traffic conditions, and can more easily influence the green production factors of cities within their plate and of cities in plate one. The fourth plate mainly receives influence from the spillover relationships it has with plate one and plate two. Most of the cities in this plate are in the eastern coastal areas, with developed economies and high eco-efficiencies. These large cities gradually form a “siphon effect”, which have a strong ability to attract green production factors from other cities. Overall, the northeastern cities play an important role in the eco-efficiency network and work as the “Bridge or agent”. Therefore, in order to boost the eco-efficiency of the whole network, the improvement of eco-efficiency in those cities would be a good place to start.

To sum up, the green production factors in the Bohai Rim area appear to gravitate to the eastern coastal areas, forming a siphon effect in their relationships with large cities. The polarization of eco-efficiency is becoming increasingly serious; that is, the spatial distribution pattern of eco-efficiency in the Bohai Rim area is “stronger in the east” and “weaker in the west”.

### 4.3. Quadratic Assignment Procedure

We found that the cities in the Bohai Rim area form a network in terms of eco-efficiency and different cities play different roles in the eco-efficiency network. However, it is still unclear how this eco-efficiency network will change over time and what factors will influence this change. In order to obtain a better understanding, we conducted a quadratic assignment procedure for analysis. Table 8 shows the results of QAP correlation analysis and the QAP regression analysis. As for the QAP regression analysis, *p* ≥ 0 indicates a probability that the regression coefficient is no less than the observed regression coefficient after the random permutation. Additionally, *p* ≤ 0 indicates the probability that the regression coefficient is no more than the observed regression coefficient after the random permutation.

From Table 8, it can be seen that geographic distance, economic difference, urbanization difference, and environmental policy difference all show a close correlation with the spatial correlation and spillover effect of eco-efficiency, and their correlations pass the 1% significance level test.

We found that the correlation coefficient for geographic distance was −0.279. Additionally, its standardized regression coefficient was −0.285, which is significant at 1%. This means that geographic distance reduces the spatial correlation and spillover effect of eco-efficiency among cities in the Bohai Rim area. Existing studies [28,29] have confirmed the positive effect of geographic proximity on technology and FDI spillovers. Therefore, the advanced green energy technologies brought by FDI are more easily transferred between neighboring cities, which in turn promotes the extension of spatial correlation and spillover networks of eco-efficiency in the Bohai Rim area.

We found that the correlation coefficient of economic difference was 0.464. Additionally, its standardized regression coefficient was 0.447, which is significant at 1%. This means that those cities which have larger economic difference are more likely to form a spatial correlation and spillover effect with eco-efficiency. In previous studies, economic development has been found to have a great influence on regional eco-efficiency [30]. However, this cannot explain why the difference in economic development poses a positive effect on the eco-efficiency network. This may be a result of the “Role model effect”. Brown and Treviño [31] studied the role model effect at an individual level and found that role model effect motivates a follower to conduct similar behaviors as those of their role model. In terms of regional government, a follower will learn from a role model with better economic development. Thus, the eco-efficiency connection between them will be stronger. On the other hand, according to the theory of international movement factors, the unevenness of regional economic development can promote the flow of production factors, such as capital and technologies, between regions. Therefore, economic gaps can affect the flow of green investment and green production technologies between cities and could thus have a positive effect on the spatial correlation and spillover effect of eco-efficiency in the Bohai Rim area.

We found that the correlation coefficient of urbanization difference was 0.254. Additionally, its standardized regression coefficient was 0.061, which is significant at 5%. This means that larger urbanization difference promotes spatial correlation and spillover effects among cities, which can also be explained by the theory international movement factors. Urbanization means that more people are flocking from rural areas to urban areas, and the increase in urban populations brings about the agglomeration of industries. Therefore, differences in urbanization between cities indicate differences in production factors, such as capital, labor, and technology, making it more likely that advanced green energy technologies will be transferred between regions. This flow of green energy technologies can have a positive impact on the spatial network of eco-efficiency in the Bohai Rim area.

We found that the correlation coefficient of environmental policy difference was −0.116. Additionally, its standardized regression coefficient was −0.052, which is significant at 1%. This means that smaller environmental policy difference among cities leads to more connections with other cities. In the literature, environmental policy has been found to play an important role in the monitoring of the environmental performance of polluters [11]. However, this kind of policy is relatively difficult to assess compared with other economic variables. Therefore, difference in environmental policy builds a barrier for cities to be able to learn from each other, considering that their regional environmental policy is different. Thus, lower difference in environmental policy will strengthen the eco-efficiency connections. More importantly, two cities with smaller differences in environmental policies are more likely to carry out regional cooperation, such as green technology innovation and collaborative governance, which will have a positive impact on the flow of production factors such as environmental investment and green technology. Therefore, smaller environmental policy differences can promote the spatial correlation and spillover effects of eco-efficiency.

The regression coefficients of technology difference and industrial structure difference are not statistically significant. However, Tang et al. [32] found that urbanization improves regional eco-efficiency through adjusting the regional industrial structure. Therefore, the information of technology difference and industrial structure difference may be represented by urbanization differences.

In summary, differences in economic development and urbanization can strengthen connections in an eco-efficiency network, while differences in geo-distance and environmental policies can weaken the connections in an eco-efficiency network. From the perspective of integrated development of eco-efficiency in the Bohai Rim area, integrated development does not mean that all cities should have similar GDP growth or urbanization progress. However, it is important to set up a role model for other cities to follow. Those cities could be provincial capitals, which comprise the “bridge” in the eco-efficiency network. As for environmental policies and transportation, the integrated development of the Bohai Rim area may require fully accessible environmental policy implementation and the development of convenient transportation across the region.

## 5. Conclusions 

Through measuring eco-efficiency, we explored the network of eco-efficiency among cities in the Bohai Rim area. This paper finds that, while eco-efficiency in the Bohai Rim area has increased over time, coinciding with increased environmental consciousness in China, the eco-efficiency of cities in the region shows a “strong east and weak west” spatial distribution pattern, with significant spatial differences. Additionally, we found that there is a “club convergence” phenomenon in terms of geographic eco-efficiency, this appears to adjust over time. In terms of the eco-efficiency network, there are no isolated cities in the network, but different cities play different roles. Through conducting a social network analysis and a block model analysis, we found that a siphon effect of “eastern coastal areas”—in terms of the green production factors in the Bohai Rim area—further aggravated the polarization of eco-efficiency. Finally, through a quadratic assignment procedure analysis, we found that the homogenization of environmental policies could further strengthen the spatial correlation and spillover effects of eco-efficiency in the Bohai Rim area. Additionally, difference in economic development and urbanization can strengthen the eco-efficiency connections, while the difference in geo-distance can weaken the eco-efficiency connections in the eco-efficiency network.

Based on the above analysis and research findings, the insights of this research are as follows: (1) It remains important to strengthen the integrated development of the Bohai Rim region. Although we have found spatial correlations in this study, many cities still have low eco-efficiency, and the network structure of eco-efficiency in the Bohai Rim area still has room for improvement. (2) We advise that the Chinese central government pays more attention to the eco-efficiency of coastal cities and provincial capitals. In the eco-efficiency network, coastal cities and provincial capitals play important roles and work as “bridges”, which means that their eco-efficiency level can significantly influence the eco-efficiencies of other cities. (3) In order to promote the eco-efficiency of the Bohai Rim area, it is essential to develop basic transportation and focus on coordinating environmental policies. In addition, central government should actively encourage communication and cooperation between cities and motivate cities with low eco-efficiency to learn from cities with high eco-efficiency. (4) The aggregation of relevant resources and elements, such as policies, funds, and technology, should be promoted, and the effect of work synergy should be amplified. (5) Green finances should be vigorously developed, and effective approaches to the development of green finances should be actively explored through innovations in financial organizations, financing models, service methods, and management systems.

This study used a social network approach and the QAP model to analyze city-level regional green development, degree of synergy, and influencing factors; our findings enrich the research on green development and spatial synergy to a certain extent, but deficiencies and imperfections remain. First of all, this paper only analyzes the spatial correlation of the green development in the urban agglomeration around the Bohai Sea, and the selected samples are limited. In the future, it is possible to compare the green development level, spatial correlation, and synergy degree of different urban agglomerations. Secondly, due to limited data, this study selected the pollutants emitted by industry. Additional contaminants should be added to the analyses in the future. Finally, the research object of this paper was cities; if the research focus was extended to a county level, then a more objective conclusion might be obtained.

## Figures and Tables

**Table 1:** Spatial correlation variables.

Factor	Variables	Measurement
Geographical proximity	Geographic distance matrix (GD)	Geographic distance matrix of city i and city j
Level of economic development	Difference matrix of economic development level (ED)	Difference in GDP per capita between city i and city j
Technological innovation level	Technological innovation difference matrix (TD)	The difference in the number of patent applications between city i and city j per 10,000 people
Urbanization level	Urbanization difference matrix (UD)	Difference in urbanization level between city i and city j
Industrial structure level	Industrial structure difference matrix (ID)	The difference in the proportion of tertiary industry structure between city i and city j
Environmental regulation level	Environmental regulation difference matrix (EPD)	The difference in the comprehensive utilization rate of general industrial solid waste between city i and city j

**Table 2:** Indicator system for the SBM model.

Layer	Variables	Definition
Inputs	Land	Construction land for cities
	Labor	Employment rate
	Water	Water consumption
	Energy	Liquefied petroleum gas
		Electricity consumption
	Capital	Capital stocks
Desirable output	GDP	GDP
Undesirable outputs	Wastewater	Industrial wastewater
	Waste gas	Industrial sulfur dioxide
	Dust	Industrial smoke dust

**Table 3:** Network features of eco-efficiency.

	2005	2006	2007	2008	2009	2010	2011	2012	2013	2014	2015	2016	2017
Correlation	1	1	1	1	1	1	1	1	1	1	1	1	1
Hierarchy	0	0	0.04	0.04	0.04	0.04	0.04	0.04	0.08	0.04	0.04	0.04	0.04
Efficiency	0.76	0.74	0.75	0.74	0.73	0.73	0.72	0.72	0.74	0.71	0.72	0.73	0.71

**Table 4:** Centrality analysis on eco-efficiency.

City	Degree Centrality		Closeness Centrality	Betweenness Centrality	City	Degree Centrality		Closeness Centrality	Betweenness Centrality
	Out	In				Out	In		
AY	13	3	81	3.59	LC	8	8	85	0.78
BYNE	3	2	106	0.00	PJ	8	5	90	1.13
BT	12	21	76	29.40	PY	8	2	88	1.43
BD	15	18	76	16.57	QHD	11	7	82	5.96
BJ	11	6	90	4.45	QD	13	32	62	70.34
BZ	4	7	90	0.30	RZ	7	4	89	0.71
CZ	9	12	82	4.06	SY	10	6	84	3.74
CY	12	8	80	7.90	SJZ	12	16	74	14.82
CD	13	5	81	8.18	TY	12	10	80	4.32
CF	15	1	79	10.82	TS	12	21	72	30.42
DL	10	14	80	11.61	TJ	12	6	91	4.59
DT	13	5	81	6.53	TL	12	2	82	4.68
DD	12	4	82	6.70	WH	19	22	65	61.89
DZ	6	9	84	2.12	WF	6	6	95	0.30
DY	13	41	54	146.17	ULCB	4	3	92	0.35
ODS	18	33	62	108.53	XZ	10	6	83	2.47
ZX	9	4	87	1.91	XT	12	17	75	17.56
HD	13	15	78	9.08	YT	16	28	65	51.09
HS	11	14	78	13.02	YQ	13	2	81	2.97
HHT	10	16	82	16.62	YK	6	5	91	0.80
HLD	12	8	81	5.59	ZZ	7	3	89	0.71
JN	12	28	67	45.10	ZJK	15	6	79	10.07
JC	12	0	82	3.01	CZ	12	2	82	3.70
LF	11	6	82	2.82	ZB	13	28	67	45.10

**Table 5:** Cluster analysis for green economic development.

Plate	City	Number
Plate one	AY, BYNE, BD, BZ, CZ, CD, DT, DZ, HD, HS, JC, LF, LC, PY, SJZ, ULCB, XZ, XT, YQ, ZJK, CZZ	21
Plate two	CY, CF, DD, FX, HLD, PJ, QHD, RZ, SY, TL, WF, YK, ZZ	13
Plate three	BT, BJ, ODS, HHT, TY, TJ	6
Plate four	DL, DY, JN, QD, TS, WH, YT, ZB	8

**Table 6:** Correlations for eco-efficiency between plates in 2017.

Plate	Reception		Spillover		Expected Internal Relationship Ratio (%)	Actual Internal Relationship Ratio (%)	Plate Feature
	In the Plate	Outside the Plate	In the Plate	Outside the Plate			
One	35	123	35	182	42.55	16.13	“Net overflow plate”
Two	30	33	30	97	25.53	23.62	“Agent plate”
Three	9	83	9	66	10.64	12.00	“Two-way overflow plate”
Four	19	199	19	93	14.90	16.96	“Main benefit plate”

**Table 7:** Density or image matrix of eco-efficiency in Bohai Rim.

Plate	Density Matrix				Image Matrix			
	One	Two	Three	Four	One	Two	Three	Four
One	0.083	0.000	0.532	0.685	0	0	1	1
Two	0.004	0.192	0.192	0.779	0	0	0	1
Three	0.484	0.026	0.300	0.063	1	0	1	0
Four	0.363	0.298	0.021	0.268	1	1	0	1

**Table 8:** Quadratic assignment procedure analysis.

Variable	Correlation Analysis		QAP Regression Analysis				
	Correlation Coefficient	*p*-Value	Non-Standardized Regression Coefficient	Standardized Regression Coefficient	*p*-Value	*p* ≥ 0	*p* ≤ 0
**GD**	−0.279	0.000	−0.241	−0.285	0.000	1.000	0.000
**ED**	0.464	0.000	0.384	0.447	0.000	0.000	1.000
**TD**	0.202	0.000	−0.005	−0.005	0.460	0.540	0.460
**UD**	0.254	0.002	0.052	0.061	0.034	0.034	0.967
**ID**	0.001	0.000	−0.022	−0.026	0.158	0.842	0.158
**EPD**	−0.116	0.000	−0.044	−0.052	0.010	0.991	0.010

## Abbreviations

Abbreviation for cities in Bohai Rim.

DMU	Abbreviation	DMU	Abbreviation	DMU	Abbreviation
Beijing	BJ	Fuxin	FX	Shenyang	SY
Anyang	AY	Handan	HD	Tianjin	TJ
Baoding	BD	Hohhot	HHT	Tongliao	TL
Baotou	BT	Huludao	HLD	Tangshan	TS
Bayannaoer	BYNE	Hengshui	HS	Taiyuan	TY
Binzhou	BZ	Jincheng	JC	Ulanchabu	ULCB
Chengde	CD	Jinan	JN	Weifang	WF
Chifeng	CF	Liaocheng	LC	Weihai	WH
Chaoyang	CY	Langfang	LF	Xingtai	XT
Cangzhou	CZH	Ordos	ODS	Xinzhou	XZ
Changzhi	CZ	Panjin	PJ	Yingkou	YK
Dandong	DD	Puyang	PY	Yangquan	YQ
Dalian	DL	Qingdao	QD	Yantai	YT
Datong	DT	Qinhuangdao	QHD	Zibo	ZB
Dongying	DY	Rizhao	RZ	Zhangjiakou	ZJK
Dezhou	DZ	Shijiazhuang	SJZ	Zizhuang	ZZ
